# Supplementary material for: Complexome profiling of the Chlamydomonas psb28 mutant reveals TEF5 as an early PSII assembly factor
Source: Plant Cell. 2025 Mar 17;37(6):koaf055. doi: 10.1093/plcell/koaf055 (PMC12164590; doi:10.1093/plcell/koaf055)
Supplement: koaf055_Supplementary_Data [file koaf055_supplementary_data.zip › psb28_interactive.zip!/plots/intensity/qualityPlots/peptides/COX5C_Cre03.g157700.t1.2.html]

### Cre03.g157700.t1.2

Trivial name: COX5C  
SeqCoverage [median]: 63.50  
Mass: 10.87 kDa  
  
 

| Sequence | MissedCleavage | Mass [Da] | wtIntensity | muIntensity | Score |
| --- | --- | --- | --- | --- | --- |
| AVYAPSEYFK | 0 | 1173.57 | 62549.42 | 93920.22 | 124.98 |
| AVYAPSEYFKYGEGASK | 1 | 1865.88 | 0.00 | 1449.36 | 43.79 |
| HFGFAK | 0 | 705.36 | 8167.80 | 10276.20 | 112.58 |
| KSALADKYK | 2 | 1022.58 | 511.82 | 0.00 | 128.52 |
| QLEEELLS | 0 | 959.48 | 14389.98 | 5728.63 | 141.78 |
| SALADKYK | 1 | 894.48 | 0.00 | 5214.32 | 115.49 |
| SQAPATAASK | 0 | 930.48 | 13001.83 | 27676.19 | 88.50 |
| YGEGASK | 0 | 710.32 | 1803.48 | 1618.06 | 83.61 |
| YIAQYYADMAR | 0 | 1363.62 | 19473.00 | 39830.95 | 158.76 |
|  |  |  | 119897.3 | 185713.9 |  |
